# Systematic review and meta-analysis of right subclavian artery variants and their correlation with cervical-thoracic clinical conditions

**DOI:** 10.1097/MD.0000000000036856

**Published:** 2024-02-23

**Authors:** Juan José Valenzuela-Fuenzalida, Mathias Orellana-Donoso, Daniela Perez-Jiménez, Emilio Farfán-Cabello, Marjorie Gold-Semmler, Alvaro Becerra-Farfan, Camila Román, Pablo Nova-Baeza

**Affiliations:** a Department of Morphology, Faculty of Medicine, Universidad Andrés Bello, Santiago, Chile; b Department of Morphology and Function, Faculty of Health, University of the Americas Santiago, Chile; c Escuela de Medicina, Universidad Finis Terrae, Santiago, Chile; d Departamento de Anatomía, Escuela de Medicina, Pontificia Universidad Católica de Chile, Santiago, Chile; e Departamento de Ciencias Química y Biológicas Facultad de Ciencias de la Salud, Universidad Bernardo O’Higgins.

**Keywords:** aberrant left subclavian artery, aberrant right subclavian artery, clinical anatomy, dysphagia lusory, Kommerell diverticulum, lusory artery, variation anatomical, variations anatomical

## Abstract

**Background:**

A high incidence of anatomical variations in the origin of the branches of the aortic arch has been reported, Nowadays, this variation is considered the most frequent in the aortic arch, its prevalence being estimated between 0.5% and 2.5% of the population. To understand its origin, knowledge of embryonic development is necessary.

**Methods:**

We searched the MEDLINE, Scopus, Web of Science, Google Scholar, Cumulative Index to Nursing and Allied Health Literature, and Latin-American literature and caribean of health sciences databases with dates ranging from their inception to June 2023. Study selection, data extraction, and methodological quality were assessed with the guaranteed tool for anatomical studies (Anatomical Quality Assurance). Finally, the pooled prevalence was estimated using a random effects model.

**Results:**

Thirty-nine studies were found that met the eligibility criteria. Twenty studies with a total of 41,178 subjects were included in the analysis. The overall prevalence of an ARSA variant was 1% (95% confidence interval = 1%–2%), the clinical findings found are that if ARSA is symptomatic it could produce changes in the hemodynamic function of the thoracocervical region in addition to other associated symptomatic complications in surrounding structures.

**Conclusions:**

ARSA can cause several types of alterations in the cervical or thoracic region, resulting in various clinical complications, such as lusory dysphagia. Hence, knowing this variant is extremely important for surgeons, especially those who treat the cervico-thoracic region. The low prevalence of ARSA means that many professionals are completely unaware of its existence and possible course and origin. Therefore, this study provides detailed knowledge of ARSA so that professionals can make better diagnoses and treatment of ARSA.

## 1. Introduction

A high incidence of anatomical variations in the origin of the branches of the aortic arch has been reported. The first variation described was the aberrant right subclavian artery (ARSA) or Lusory artery made by Doctor François-Joseph Hunauld in the year 1735.^[1]^ Nowadays, this variation is considered the most frequent in the aortic arch, its prevalence being estimated between 0.5% and 2.5% of the population.^[2–4]^ To understand its origin, knowledge of embryonic development is necessary. Hegazy^[5]^ reported that the embryonic origin of the subclavian artery is as follows: the stem arises from the right aortic arch artery, while its distal part develops from the right dorsal aorta (the part between the fourth arch and the seventh intersegmental artery) and is completed by the right seventh intersegmental artery.^[5,6,7]^ the anomalous origin of the right subclavian artery can be explained by the involution of the fourth right aortic arch, with the persistence of the seventh right intersegmental artery, which maintains its connection with the dorsal aorta. In 5% of cases, this condition is symptomatic. Lusoria dysphagia (LD) is observed in 34%, dyspnea in 25%, chest pain in 16%, cough in 8%, and claudication of the corresponding upper limb in 5% of the symptomatic cases.^[8]^ In 60% of cases, ARSA may be associated with a Kommerell’s diverticulum (KD), an aneurysm that originates from the descending thoracic aorta that may be relevant in surgical approaches to treat LD.^[7]^ Among the complications associated with ARSA, we find aortic dissection, recurrent pneumonia, and obstructive emphysema.^[9,10]^ Different radiological tools are used to identify ARSA, with barium esophagram being the most indicated modality to detect LD, followed by computed tomography, magnetic resonance angiography, and Doppler ultrasound.^[11]^ The LD treatment can be conservative or surgical, depending on the patient’s symptoms. Invasive treatment is chosen when the patient reports clinical discomfort and/or the presence of a KD larger than 30 mm.^[12,13]^ From a historical point of view, AL is described as an ARSA that is associated with compression of the esophagus. However, within the articles found, the term LD is used for any artery that generates compression of the esophagus, manifesting symptoms related to dysphagia. Such is the case of aberrant left subclavian artery, which presented a retroesophageal course pattern compressing the esophagus and generating symptoms that correspond to LD.^[14–20]^ And even more, the study by Quintero-Pérez et al^[21]^ describes an aberrant right internal carotid artery with a retropharyngeal course pattern, which would generate compression of the pharynx and thereby result in symptoms associated with LD. This evidences the need to clarify the proper use of the term Lusory artery when used to describe the vessel that compresses the esophagus.

The objective reports the prevalence, anatomy, and clinical characteristics of ARSA variants and how they could be associated with pathologies of the cervical and thoracic region.

## 2. Methods

### 2.1. Protocol and registration

This systematic review and meta-analysis were performed and reported according to the Preferred Reporting Items for Systematic Reviews and Meta-Analyses statement.^[22]^

### 2.2. Eligibility criteria

Studies on the presence of ARSA and its association with any clinical condition were considered eligible for inclusion if the following criteria were fulfilled for population, outcomes, and studies, respectively: sample of dissections or images of the ARSA; ARSA prevalence, variants, and correlation with pathologies of the thoracic and neck region (additionally, anatomical variants were classified and described based on normal anatomy and classifications proposed in the literature); and research articles, research reports, or original research published in English in peer-reviewed journals and indexed in some of the databases reviewed. Conversely, the exclusion criteria were as follows: a population of animal studies; studies that performed analysis of variants of the subclavian artery that did not result in its origin or in its course before reaching the upper limb; studies consisting of letters to the editor or comments.

### 2.3. Electronic search

We systematically searched MEDLINE (via PubMed), Web of Science, Google Scholar, the Cumulative Index to Nursing and Allied Health Literature, and Scopus with dates ranging from inception to June 2023.

The search strategy included a combination of the following terms: “subclavian aberrant artery” (no MeSH), “aberrant right subclavian artery” (no MeSH), “lusory artery” (no MeSH), “aberrant left subclavian artery” (no MeSH), “variation anatomical” (no MeSH), “clinical anatomy” (no MeSH), and “kommerell diverticulum” (no MeSH) “dysphagia lusory” (no MeSH) “variations anatomical” (no MeSH) using the Boolean connectors “AND,” “OR,” and “NOT.” The search strategies for each database are available in the (Supplementary Table S1, Supplemental Digital Content, http://links.lww.com/MD/L362).

### 2.4. Study selection

Two authors (JJV-F and PN-B) independently screened the titles and abstracts of references retrieved from the searches. We obtained the full text for references that either author considered to be potentially relevant. We involved a third reviewer (DP-J) if consensus could not be reached.

### 2.5. Data collection process

Two authors (CR and MO-D) independently extracted data on the outcomes of each study. The following data were extracted from the original reports: authors and year of publication, type of study and number of subjects, incidence and characteristics, statistical values and characteristics, geographic region, and gender.

### 2.6. Assessment of the methodological quality of the included studies

**Figure 1.**
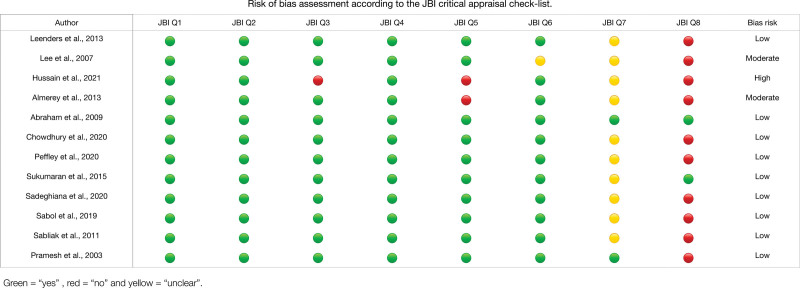
Risk of bias assessment according to the JBI critical appraisal checklist. Each article was assessed using 8 questions by selecting answers “yes,” “unclear,” “no,” or “not applicable.” Articles were evaluated using the criteria: low risk of bias—>70% “yes” score, moderate risk of bias—50% to 69% “yes” score, and high risk of bias—<49% “yes” score. Two authors independently applied this tool to each case report to reach an overall appraisal judgment with supporting justifications for each article. JBI = Joanna Briggs Institute.

Quality assessment was performed using the methodological quality assurance tool for anatomical studies (Anatomical Quality Assurance) proposed by the International Evidence-Based Anatomy Working Group^[23]^ (Table 1). Data extraction and quality assessment were independently performed by 2 reviewers (JJV-F and DP-J). For case studies, 2 authors (MO-D and PN-B) independently analyzed the studies and improved the Joanna Briggs Institute critical appraisal checklist for case reports (last amended in 2017) (Fig. 1).^[51]^ We involved a third reviewer (EF-C) if a consensus could not be reached. The agreement rate between the reviewers was calculated using kappa statistics.

**Table 1:** Aqua checklist.

References	Study design	Domain 1				Domain 2					Domain 3						Domain 4					Domain 5				
		1	2	3	4	5	6	7	8	9	10	11	12	13	14	15	16	17	18	19	20	21	22	23	24	25
Natsis et al 2016^[24]^	Observational	Y	Y	Y	Y	Y	Y	Y	N	Y	Y	Y	N	Y	Y	N	Y	Y	Y	Y	N	Y	Y	Y	NA	N
Morlando et al 2021^[25]^	Retrospective study	Y	Y	Y	Y	Y	N	Y	Y	N	Y	Y	Y	Y	Y	Y	Y	Y	N	N	Y	N	Y	Y	NA	Y
Maya et al 2016^[26]^	Multicenter study	Y	Y	Y	Y	Y	Y	Y	N	Y	Y	Y	N	Y	Y	N	Y	Y	Y	Y	N	Y	Y	Y	NA	N
Behram et al 2021^[27]^	Retrospective study	Y	Y	Y	Y	Y	N	Y	Y	N	Y	Y	Y	Y	Y	Y	Y	Y	N	N	Y	N	Y	Y	NA	Y
Jan et al 2018^[28]^	Observational	Y	Y	Y	Y	Y	Y	Y	N	Y	Y	Y	N	Y	Y	N	Y	Y	Y	Y	N	Y	Y	Y	NA	N
Dueppers et al 2020^[29]^	Retrospective study	Y	Y	Y	Y	Y	N	Y	Y	N	Y	Y	Y	Y	Y	Y	Y	Y	N	N	Y	N	Y	Y	NA	Y
Dieffenbach et al 2019^[30]^	Retrospective study	Y	Y	Y	Y	Y	Y	Y	N	Y	Y	Y	N	Y	Y	N	Y	Y	Y	Y	N	Y	Y	Y	NA	N
Chen et al 2021^[31]^	Retrospective study	Y	Y	Y	Y	Y	N	Y	Y	N	Y	Y	Y	Y	Y	Y	Y	Y	N	N	Y	N	Y	Y	NA	Y
Yusuf et al 2007^[32]^	Retrospective study	Y	Y	Y	Y	Y	Y	Y	N	Y	Y	Y	N	Y	Y	N	Y	Y	Y	Y	N	Y	Y	Y	NA	Y
Muraoka et al 2017^[33]^	Retrospective study	Y	Y	Y	Y	Y	N	Y	Y	Y	Y	Y	Y	Y	Y	Y	Y	Y	N	N	Y	N	Y	Y	NA	Y
Nayak et al 2006^[34]^	Observational	Y	Y	Y	Y	Y	Y	Y	N	Y	Y	Y	N	Y	Y	N	Y	Y	Y	Y	N	Y	Y	Y	NA	N
Chavda et al 2014^[35]^	Observational	Y	Y	Y	Y	Y	N	Y	Y	N	Y	Y	Y	Y	Y	N	Y	Y	N	N	Y	N	Y	Y	NA	Y
Acar et al 2013^[36]^	Observational	Y	Y	Y	Y	Y	Y	Y	N	Y	Y	Y	N	Y	Y	Y	Y	Y	Y	Y	N	Y	Y	Y	NA	N
Arpasi et al 2000^[37]^	Observational	Y	Y	Y	Y	Y	N	Y	Y	N	Y	Y	Y	Y	Y	Y	Y	Y	Y	N	Y	N	Y	Y	NA	Y
Bhatia et al 2005^[38]^	Observational	Y	Y	Y	Y	Y	Y	Y	N	Y	Y	Y	N	Y	Y	N	Y	Y	Y	Y	N	Y	Y	Y	NA	N
Carpenter et al 1997^[39]^	Observational	Y	Y	Y	Y	Y	N	Y	Y	N	Y	Y	Y	Y	Y	Y	Y	Y	N	N	Y	N	Y	Y	NA	Y
Chen et al 2013^[40]^	Observational	Y	Y	Y	Y	Y	Y	Y	N	Y	Y	Y	N	Y	Y	N	Y	Y	Y	Y	N	Y	Y	Y	NA	N
Demertzis et al 2010^[41]^	Observational	Y	Y	Y	Y	Y	N	Y	N	N	Y	Y	Y	Y	Y	Y	Y	Y	N	N	Y	N	Y	Y	NA	Y
Dumfarth et al 2015^[42]^	Observational	Y	Y	Y	Y	Y	Y	Y	N	Y	Y	Y	N	Y	Y	N	Y	Y	Y	Y	N	Y	Y	Y	NA	N
Ergun et al 2013^[43]^	Observational	Y	Y	Y	Y	Y	N	Y	Y	N	Y	Y	Y	Y	Y	Y	Y	Y	N	N	Y	N	Y	Y	NA	Y
Faggioli et al 2013^[44]^	Observational	Y	Y	Y	Y	Y	Y	Y	N	Y	Y	Y	N	Y	Y	N	Y	Y	Y	Y	N	Y	Y	Y	NA	N
Gielecki et al 2004^[45]^	Observational	Y	Y	Y	Y	Y	N	Y	Y	N	Y	Y	Y	Y	Y	Y	Y	Y	N	N	Y	N	Y	Y	NA	Y
Karacan et al 2014^[46]^	Observational	Y	Y	Y	Y	Y	Y	Y	N	Y	Y	Y	N	Y	Y	N	Y	Y	Y	Y	N	Y	Y	Y	NA	N
Kondori et al 2016^[47]^	Observational	Y	Y	Y	Y	Y	N	Y	N	N	Y	Y	Y	Y	Y	Y	Y	Y	N	N	Y	N	Y	Y	NA	Y
Li et al 2011^[48]^	Observational	Y	Y	Y	Y	Y	Y	Y	N	Y	Y	Y	N	Y	Y	N	Y	Y	Y	Y	N	Y	Y	Y	NA	Y
Liu et al 2009^[49]^	Observational	Y	Y	Y	Y	Y	N	Y	Y	N	Y	Y	Y	Y	Y	Y	Y	Y	N	N	Y	N	Y	Y	NA	Y
Prince et al 1996^[50]^	Observational		Y	Y	Y	Y	Y	Y	N	Y	Y	Y	N	Y	Y	Y	Y	Y	Y	Y	N	Y	Y	Y	NA	N

Domains and questions: Domain 1: objective(s) and subject characteristics. Was (Were) the objective(s) of the study clearly defined? Was (Were) the chosen subject sample(s) and size appropriate for the objective(s) of the study? Are the baseline and demographic characteristics of the subjects (age, sex, ethnicity, healthy or diseased, etc) appropriate and clearly defined? Could the method of subject selection have in any way introduced bias into the study? Domain 2: study design. Does the study design appropriately address the research question(s)? Were the materials used in the study appropriate for the given objective(s) of the study? Were the methods used in the study appropriate for the given objective(s) of the study? Was the study design, including methods/techniques applied in the study, widely accepted or standard in the literature? If “no,” are the novel features of the study design clearly described? Could the study design have in any way introduced bias into the study? Domain 3: methodology characterization. Are the methods/techniques applied in the study described in enough detail for them to be reproduced? Was the specialty and the experience of the individual(s) performing each part of the study (such as cadaveric dissection or image assessment) clearly stated? Are all the materials and methods used in the study clearly described, including details of manufacturers, suppliers etc? Were appropriate measures taken to reduce inter- and intra-observer variability? Do the images presented in the study indicate an accurate refection of the methods/techniques (imaging, cadaveric, intraoperative, etc) applied in the study? Could the characterization of methods have in any way introduced bias into the study? Domain 4: descriptive anatomy. Were the anatomical definition(s) (normal anatomy, variations, classifications, etc) clearly and accurately described? Were the outcomes and parameters assessed in the study (variation, length, diameter, etc) appropriate and clearly defined? Were the figures (images, illustrations, diagrams, etc) presented in the study clear and understandable? Were any ambiguous anatomical observations (i.e., those likely to be classified as “others”) clearly described/depicted? Could the description of anatomy have in any way introduced bias into the study? Domain 5: reporting of results. Was the statistical analysis appropriate? Are the reported results as presented in the study clear and comprehensible, and are the reported values consistent throughout the manuscript? Do the reported numbers or results always correspond to the number of subjects in the study? If not, do the authors clearly explain the reason(s) for subject exclusion? Are all potential confounders reported in the study, and subsequently measured and evaluated, if appropriate? Could the reporting of results have in any way introduced bias into the study? (Henry et al^[23]^).

### 2.7. Statistical methods

The data extracted from the meta-analysis were interpreted through calculation of the prevalence of the APBJ variants using JAMOVI software.^[52]^ The DerSimonian–Laird model with a Freeman–Tukey double arcsine transformation was used to combine the summary data. In addition, a random effects model was used because the APBJ prevalence data were very heterogeneous. The degree of heterogeneity between included studies was assessed using the chi² test and the heterogeneity (*I*²) statistic. For the χ² test, the *P* value proposed by the Cochrane collaboration was considered significant when it was <0.10. Values of the *I*² statistic were interpreted with a 95% confidence interval in the following way: 0% to 40% might not be important, 30% to 60% might indicate moderate heterogeneity, 50% to 90% might represent substantial heterogeneity, and 75% to 100% could represent a significant amount of heterogeneity.

## 3. Results

### 3.1. Characteristics of included studies

**Figure 2.**
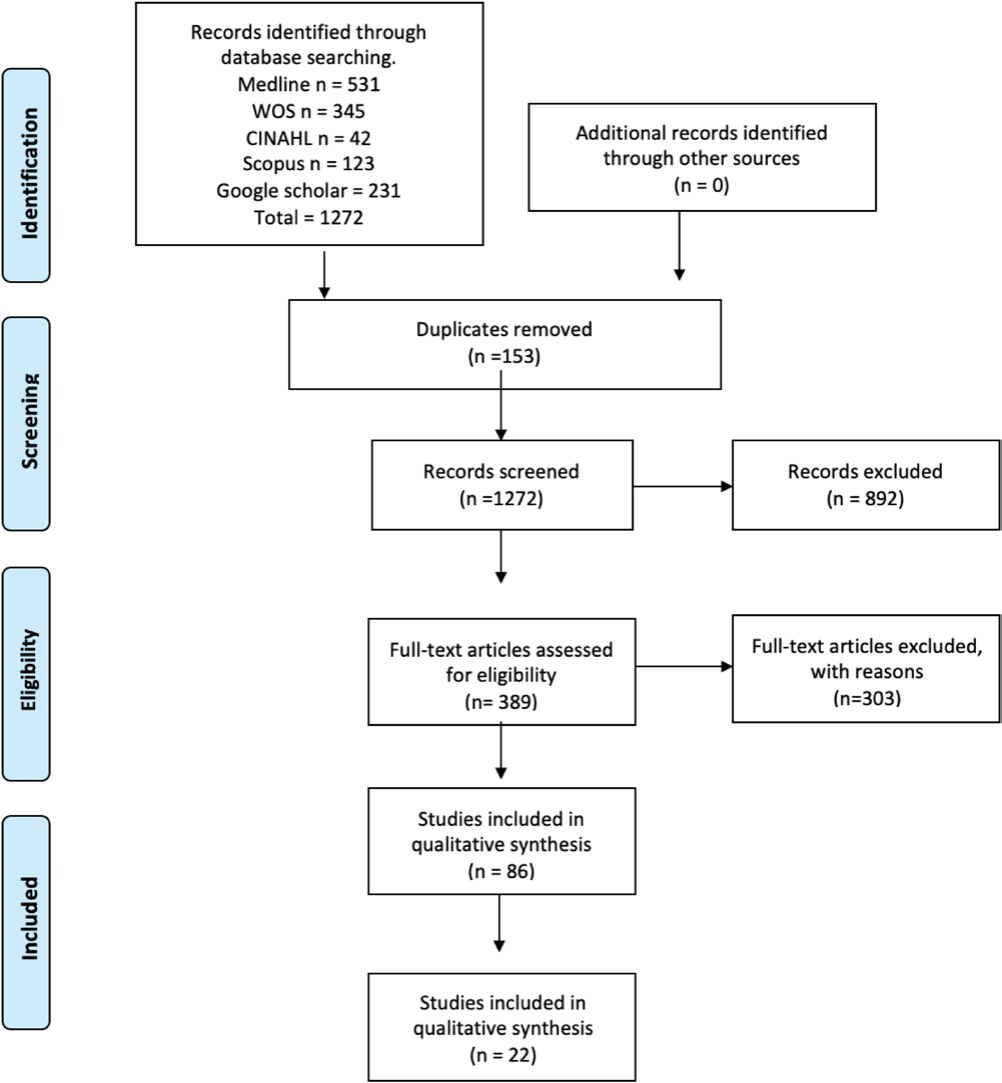
Search flowchart. CINAHL = Cumulative Index to Nursing and Allied Health Literature, WOS = Web of Science.

Within the search results based on the inclusion and exclusion criteria proposed by this research team, 1272 studies that met the criteria were found, of which 86 were analyzed in full text, 59 were case studies, and 27 were experimental studies. The inclusion of case studies was based on analyzing whether there were samples with important characteristics that adapted to our review or some special type in relation to this selection criterion; 47 case reports that did not meet the criteria were eliminated. The aforementioned criteria are detailed in Supplementary Table 2, Supplemental Digital Content, http://links.lww.com/MD/L365 and Figure 2. With a total of 39 included studies, for the 27 studies that had an N > 1,^[24–50]^ the following data were collected: the total number of subjects included in the studies was 41,178 with an average ratio of 1.525:1 regarding the sex of the sample; 12 studies did not indicate the sex of their sample^[25,27,31,34,35,37,38,45,47–50]^; one study by Yusuf et al^[32]^ only indicates the sex of the sample that did present the anatomical variant and consisted of 10 men and 17 women, respectively; and, finally, 14 studies^[24,26,28–30,33,39–44,46]^ show that the cumulative data for males was 2964/5393, which is equivalent to 54.9%, and cumulative data for females was 2429/5393, which is equivalent to 45.1%. Finally, regarding the geographical distribution of the included studies,^[24–50,53–64]^ in 13 studies, the sample was European, which is equivalent to 33.3% of the included studies; in 12 studies, the sample was from North America, which is equivalent to 30.8% of the included studies; and, finally, 14 studies had a sample from Asia, which is equivalent to 35.9% of the studies included in this review (Table 2).

**Table 2:** Characteristics of studies included.

	Type of study and N	Incidence and characteristics	Statistical values and characteristics	Geographic region	Gender
Chowdhury et al 2020^[63]^	Case study (1)	ARSA with retroesophageal trajectory (100%)	Does not present	USA	Female (100%) 58 yr
Peffley et al 2020^[61]^	Case study (1)	ARSA with retroesophageal trajectory (100%)	Does not present	Canada	Female (100%) 40 yr
Natsis et al 2016^[24]^	Observational (267)	ARSA in 6 cadavers (2.2%), 4 women (2.8%), and 2 men (1.6%). Retroesophageal route in 83% and interesophageal tracheal route in 16.7% (2.2%).	Does not present	Greece	267 corpses in formaldehyde: 126 male and 141 female Middle ages: 59 ± 13 yr
Morlando et al 2021^[25]^	Retrospective study (50)	50 fetuses with ARSA with postnatal confirmation. 46 with isolated presentation (92%) and 4 in association with malformations. 1 with trisomy 21 (2%) (100%)	Does not present	Italy	50 Fetuses (does not specify gender)
Maya et al 2016^[26]^	Multicenter study (63)	63 fetuses with ARSA: 36 with isolated ARSA and normal chromosome microarray analysis (57% female). 10 with minor signs of aneuploidy (45% female). 17 with ARSA and major signs of abnormalities (64% female) (100%).	Does not present	Israel	63 fetuses 34 female (54%) 29 male (46%)
Jan et al 2018^[28]^	Observational study (1737)	15 cases with isolated ARSA (0.86%)	Newborns with ARSA have a 21% higher incidence of IUGR (*P* = .061). Newborns with ARSA have a higher incidence of feeding difficulties, 36% compared to 20% in normal newborns (*P* = .264).	Taiwan	15 cases with ARSA of 1737 full-term newborns 900 male (52%) 837 female (48%)
Dueppers et al 2020^[29]^	Retrospective study (16)	Symptomatic ARSA with or without KD (100% patients studied)	Does not present	Germany	8 patients. 4 male 4 female Middle ages: 63 ± 14 yr
Dieffenbach et al 2019^[30]^	Retrospective study (10)	Symptomatic ARSA with or without KD. (100% patients studied)	Does not present	USA	10 patients. 1 male 9 female Middle ages: 48 ± 12 yr
Chen et al 2021^[31]^	Retrospective study (13,690)	ARSA Incidence of 0.69% (95/13,690). 66.32% (63/95) with isolated ARSA. 33.68% (32/95) ARSA associated with malformations.	Does not present	China	13,690 single pregnancies Gestational age: 16 wk + 0 d to 38 wk + 5
Behram et al 2021^[27]^	Retrospective study (11,666)	ARSA in 140 fetuses (1.2%). Isolated in 47.1% (66/140) and associated with malformations in the remaining 52.9% (74/140). With chromosomal abnormalities in 17.8% (25/140). Of these: trisomy 21 in 11.4% (16/140), Di George syndrome in 1.4% (2/140), Turner syndrome in 1.4% (2/140), and trisomy 18 in 2.8% (4/140).	Does not present	Turkey	11,666 fetuses Mean gestational age of 22.3 ± 4.5 wk
Yusuf et al 2007^[32]^	Retrospective study (7513)	Retroesophageal ARSA in 27 exams (0.36%, 95% CI: 0.22%–0.50%) 1 patient with KD	Does not present	USA	7513 US exams. Only the sex of those with the variant was reported. 10 male and 17 female Median age: 58 yr (range 23–81 yr)
Sukumaran et al 2015^[59]^	Case study	ARSA with course to the right between the trachea and the esophagus 100% incidence	Does not present	India	1 male Age: 35 yr
Sadeghian et al 2020^[62]^	Case study	ARSA with dysphagia 100% incidence	Does not present	USA	1 male Age: 52 yr
Sabol et al 2019^[60]^	Case study	Right aortic arch, aberrant left subclavian artery aortic coarctation 100% incidence	Does not present	Slovakia	1 female Age: 51 yr
Sabljak et al 2011^[56]^	Case study	Hypopharyngeal and cervical esophageal carcinoma with coexistence of ARSA 100% incidence	Does not present	Serbia	1 female Age: 65 yr
Pramesh et al 2003^[53]^	Case study	Esophageal carcinoma with coexistence of ARSA 100% incidence	Does not present	India	1 female Age: 52 yr
Muraoka et al 2017^[33]^	Retrospective study (10)	Aberrant left subclavian artery with or without stenosis 100% incidence	Does not present	Japan	10 fetuses: 6 male 4 female Age: 24–39 wk
Leenders et al 2013^[57]^	Case study	Occluded aneurysmal ARSA 100% incidence	Does not present	The Netherlands	1 female Age: 51 yr
Lee et al 2006^[54]^	Case study	ARSA with KD 100% incidence	Does not present	USA	1 male Age: 84 yr
Hussain et al 2021^[64]^	Case study	ARSA with aberrant right retroesophageal aneurysm 100% incidence	Does not present	USA	1 male Age: 27 yr
Almerey et al 2013^[58]^	Case study	ARSA with internal thoracic artery aneurysm 100% incidence	Does not present	USA	1 female Age: 54 yr
Abraham et al 2009^[55]^	Case study	ARSA 100% incidence	Does not present	India	3 male Age: 14–58 yr
Chavda et al 2014^[35]^	Observational study (70)	1 cadaver with ARSA	Does not present	India	No reported
Nayak et al 2006^[34]^	Observational study (61)	1 cadaver with ARSA	Does not present	India	No reported
Acar et al 2013^[36]^	Observational study (94)	5 subjects in their MRI	Does not present	Turkey	32 male and 62 female
Arpasi et al 2000^[37]^	Observational study (49)	2 subjects in their MRI with ARSA	Does not present	USA	No reported
Bhatia et al 2005^[38]^	Observational study (545)	47 subjects with ARSA	Does not present	China	No reported
Carpenter et al 1997^[39]^	Observational study (28)	1 subject with ARSA	Does not present	USA	14 male and 14 female
Chen et al 2013^[40]^	Observational study (198)	10 subjects with ARSA	Does not present	China	125 male 73 female
Demertzis et al 2010^[41]^	Observational study (92)	2 subjects with ARSA	Does not present	Suiza	79 males 13 females
Dumfarth et al 2015^[42]^	Observational study (556)	12 subjects with ARSA	Does not present	USA	395 males 261 females
Ergun et al 2013^[43]^	Observational study (1001)	11 subjects with ARSA	Does not present	Turkey	519 males 482 females
Faggioli et al 2013^[44]^	Observational study (214)	3 subjects with ARSA	Does not present	Italy	119 males 105 females
Gielecki et al 2004^[45]^	Observational study (103)	3 subjects with ARSA	Does not present	Poland	No reported
Karacan et al 2014^[46]^	Observational study (1000)	10 subjects with ARSA	Does not present	Turkey	610 males 390 females
Kondori et al 2016^[47]^	Observational study (226)	5 subjects with ARSA	Does not present	Iran	No reported
Li et al 2011^[48]^	Observational study (1300)	14 subjects with ARSA	Does not present	China	Not reported
Liu et al 2009^[49]^	Observational study (600)	12 subjects with ARSA	Does not present	China	Not reported
Prince et al 1996^[50]^	Observational study (19)	1 subject with ARSA	Does not present	USA	Not reported

ARSA = aberrant right subclavian artery, CI = confidence interval, KD = Kommerell’s diverticulum.

### 3.2. Variant features

**Figure 3.**
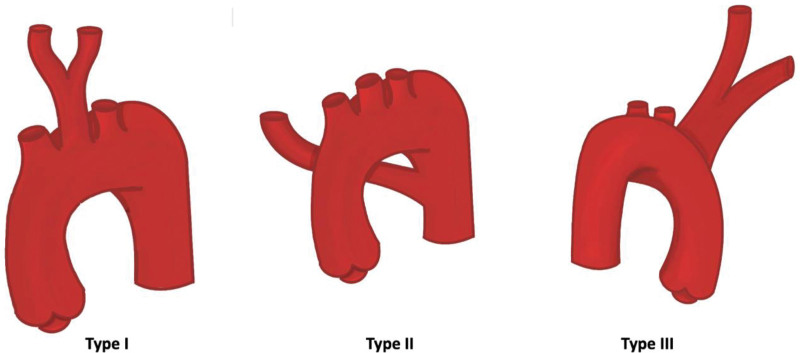
Types of origin of ARSA. Type I corresponds to an ARSA that arises as the last branch of the aortic arch, maintaining the origin of the other branches of this arch. Type II corresponds to an aberrant left subclavian artery associated with a bicarotid trunk, formed by both common carotid arteries. Type III is described as a mirror pattern of type I, with a right aortic arch from which arises a subclavian artery with a retroesophageal course that passes posterior to the 2 carotids and the right subclavian artery. ARSA = aberrant right subclavian artery.

**Figure 4.**
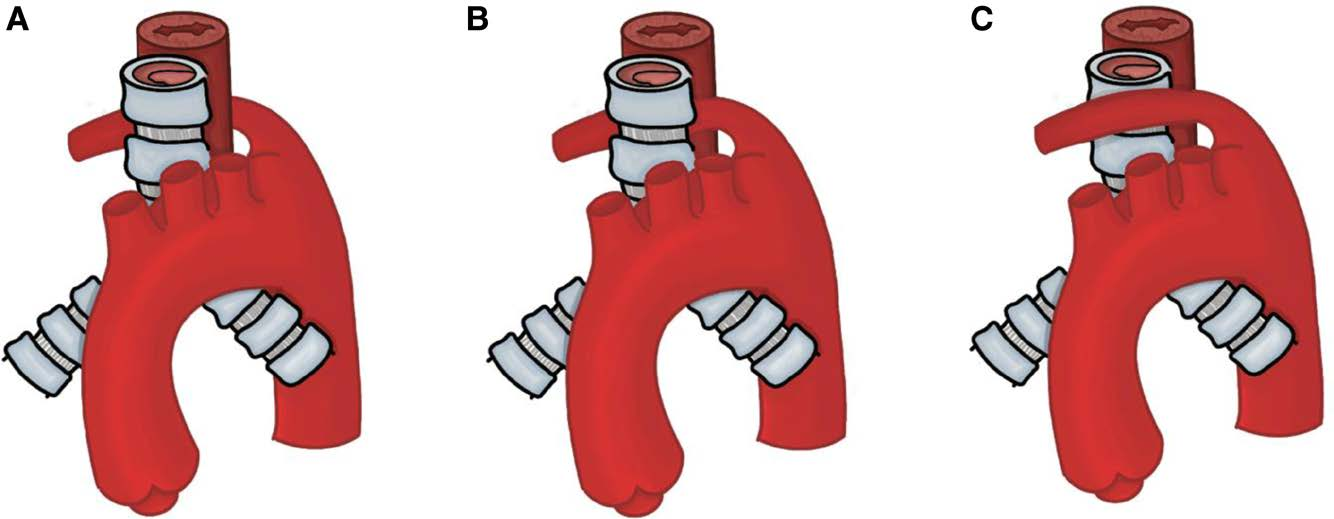
ARSA route types. (A) Retroesophageal course. (B) Course between the trachea and the esophagus. (C) Anterior course of the trachea. ARSA = aberrant right subclavian artery.

The types of variations described in the analyzed articles can be divided into variations in the origin of the subclavian artery (Fig. 3) or also in variations in its route (Fig. 4). In the origin variations, 3 types were found. Type I corresponds to an ARSA that arises as the last branch of the aortic arch, maintaining the origin of the other branches of this arch.^[49]^ Type II corresponds to an aberrant left subclavian artery associated with a bicarotid trunk, formed by both common carotid arteries.^[41]^ Type III is described as a mirror pattern of type I, with a right aortic arch from which a subclavian artery arises with a retroesophageal course that passes posteriorly to the 2 carotid arteries and the right subclavian artery.^[48]^ The first is the retroesophageal route, where the artery is directed cephalad passing posterior to the esophagus; this type is the most common.^[65]^ In the second type, the artery runs between the esophagus and the trachea. And in the third type, the artery runs anterior to the trachea.^[66]^ For the included studies that could be classified according to their origin with respect to what was proposed by Williams et al,^[66]^ 7 studies^[32,54–56,59,63,64]^ reported the ARSA variant, showing the characteristics according to origin in 6 studies,^[32,55,56,59,63,65]^ the variant is classified as type I in its origin, while 2 studies^[54,55]^ showed that ARSA was classified as a different type of variant according to its origin and the classification. While 11 studies^[24,29,32,55–57,59,61–64]^ in their description classified the route as type I, for the classification of type II, route 2 studies^[24,59]^ presented this pattern; finally, for the type III route classification, no studies were presented (Tables 3 and 4).

**Table 3:** Classification to ARSA according to your origin.

Author and year	N	According to origin: type I	According to origin: type II	According to origin: type III	Other	Does not report description	Observation
Chowdhury et al 2020^[63]^	1	1	-	-	,	-	-
Yusuf et al 2007^[32]^	27	27	-	-	-	-	-
Sukumaran et al 2015^[59]^	1	1	-	-	-	-	-
Sabljak et al 2011^[56]^	1	1	-	-	-	-	-
Lee et al 2006^[54]^	-	-	-	-	1	-	Origin in KD
Hussain et al 2021^[64]^	1	1	-	-	-	-	Origin in Ao arch aneurysm
Abraham et al 2009^[55]^	3	1	-	-	2	-	Origin in KD

ARSA = aberrant right subclavian artery, KD = Kommerell’s diverticulum.

**Table 4:** Classification to ARSA according to your route.

Author and year	N	Retroesophageal course: type I	Course between the trachea and the esophagus: type II	Anterior course of the trachea: type III	Other	Does not report description	Observation
Chowdhury et al 2020^[63]^	1	1	-	-	-	-	-
Peffley et al 2020^[61]^	1	1	-	-	-	-	-
Natsis et al 2016^[24]^	6	5	1	0	0	-	Type I in 83% and type II in 16.7%.
Dueppers et al 2020^[29]^	8	8	-	-	-	-	-
Yusuf et al 2007^[32]^	27	27	-	-	-	-	-
Sukumaran et al 2015^[59]^	1	-	1	-	-	-	-
Sadeghian et al 2020^[62]^	1	1	-	-	-	-	-
Sabljak et al 2011^[56]^	1	1	-	-	-	-	-
Leenders et al 2013^[57]^	1	1	-	-	-	-	-
Hussain et al 2021^[64]^	1	1	-	-	-	-	-
Abraham et al 2009^[55]^	3	1	-	-	-	2	-

ARSA = aberrant right subclavian artery.

### 3.3. Prevalence and risk of bias

**Figure 5.**
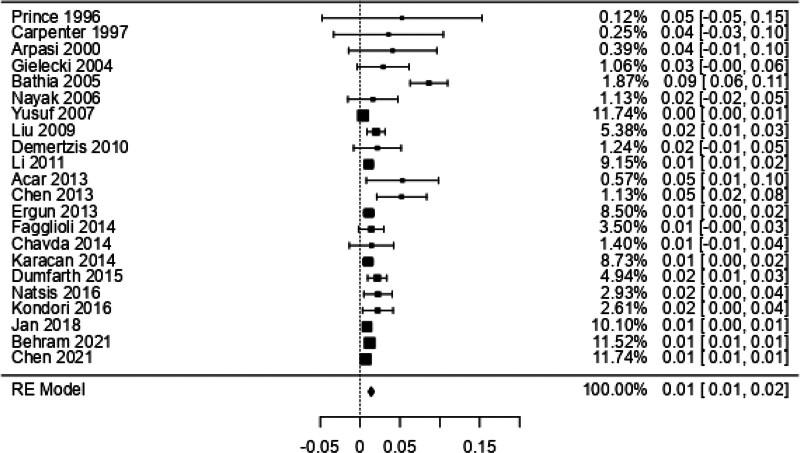
Forest plot prevalence ARSA. ARSA = aberrant right subclavian artery.

**Figure 6.**
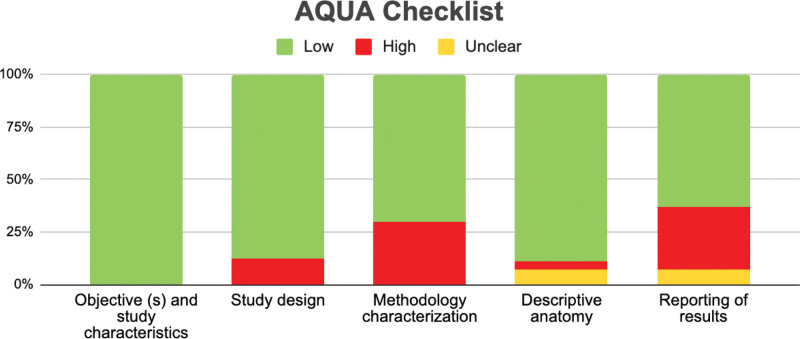
Risk of bias included studies. AQUA = Anatomical Quality Assurance.

For the ARSA prevalence meta-analysis, 22 studies,^[30,39]^ with a prevalence of 1% and a confidence interval of 1% to 2%, showing a heterogeneity of *I*² = 83.6%, which is high, were included (Figure 5 and Table 5). Within the 12 case studies, only 1 article^[64]^ presented a high risk of bias, while the other studies fluctuated between low and moderate risk of bias. For the risk of bias of studies with n > 1 (Anatomical Quality Assurance checklist) in the first domain that covers the research objectives, for the previous one, all the studies showed a low risk of bias; in the second domain that evaluates the study design, only 2 studies showed a high risk of bias; for the third domain that evaluates the methodological characteristics of the study, 8 studies presented a high risk of bias; for the fourth domain that covers the anatomical description of the studies, only 1 presented a high risk of bias, and 2 studies presented as unclear; finally, the fifth domain assessing outcome reported 8 studies with a high risk of bias, and 2 studies were unclear (Fig. 6).

**Table 5:** Prevalence to ARSA.

Author and year	Total N	Prevalence
Prince et al 1996^[50]^	19	1
Carpenter et al 1997^[39]^	28	1
Arpasi et al 2000^[37]^	49	2
Gielecki et al 2004^[45]^	103	3
Bhatia et al 2005^[38]^	545	47
Nayak et al 2006^[34]^	62	1
Yusuf et al 2007^[32]^	7513	28
Liu et al 2009^[49]^	600	12
Demertzis et al 2010^[41]^	92	2
Li et al 2011^[48]^	1300	14
Acar et al 2013^[36]^	94	5
Chen et al 2013^[40]^	192	10
Ergun et al 2013^[43]^	1001	11
Faggioli et al 2013^[44]^	214	3
Chavda et al 2014^[35]^	70	1
Karacan et al 2014^[46]^	1000	10
Dumfarth et al 2015^[42]^	556	12
Natsis et al 2016^[24]^	267	6
Kondori et al 2016^[47]^	226	5
Jan et al 2018^[28]^	1737	15
Behram et al 2021^[27]^	11,666	140
Chen et al 2021^[31]^	13,690	95

ARSA = aberrant right subclavian artery.

### 3.4. Clinical considerations

The most common symptoms associated with ARSA occur more commonly after the age of 40 because at this age, the aortic wall becomes more atherosclerotic and therefore more rigid. Among the most common associated symptoms are dysphagia, cough, noisy breathing, chest pain, and lower respiratory tract infections. Associated with this symptomatology, more serious complications can occur, such as aortic dissection that can trigger a complete rupture of the aortic wall, generating mediastinal hemorrhage, which can be fatal; recurrent pneumonia and obstructive emphysema have also been described.^[8,9]^ Therefore, it is evident that the symptoms of ARSA plus LD must be identified so that a diagnosis can occur. According to one article, when the presence of lustful dysphagia is suspected, the diagnosis is performed through a computerized axial tomography^[57]^; in contrast, in another article, it is recommended to start the diagnosis with an esophagram.^[29]^ In 5 studies, we found that the procedure to be performed is a surgical intervention to alleviate the symptom of lustful dysphagia.^[30,32,61,63,67]^ It should be noted that 1 study emphasizes that if the patient is not a candidate or does not approve of the surgical intervention, endoscopic dilation can be performed, which is the best option due to its availability, ease of use, and lower risk and complication rate.^[62]^ There are 3 types of KD: diverticulum in a left aortic arch with ARSA, diverticulum in a right aortic arch with aberrant left subclavian artery, and diverticulum at the aorticoductal junction.^[12,68–70]^ Of the 3 types, the second is the one that describes the majority of the cases found in this review. The foregoing was associated in the following way within the included studies: 14 studies mention that ARSA is asymptomatic in patients, but in cases of manifesting symptoms, the most common is LD together with dyspnea.^[24,25,27,29,32,53,56,58,59,61–64,67]^ Within the articles described, 2 of them emphasize that ARSAs with a retroesophageal and retrotracheal course are prone to manifest compression symptoms due to the proximity of the anatomical structures.^[24,58]^

## 4. Discussion

This systematic review and meta-analysis aimed to report the characteristics and prevalence of ARSA. The prevalence calculation of the studies that met the inclusion criteria showed that the presence of ARSA is very low in relation to the included studies; We have also been able to realize that ARSA should not always be associated with LD, and we have been able to demonstrate that in very few cases of the included studies there was a direct relationship between both anomalies. Furthermore, among the main clinical findings found is that if ARSA is symptomatic, it could produce changes in the hemodynamic function of the thoracocervical region in addition to other associated symptomatic complications in surrounding structures, so in the presence of symptoms and with structural changes or important hemodynamics, surgical management is recommended.

Regarding other review articles that have related anatomical variants of the ARSA and their clinical considerations, our review presents a detailed anatomical approach and classifications of the ARSA and how this influences different clinical conditions in the thoracic and cervical region. We also add updated terminology of the anatomical structures that make up the origin and course of ARSA; in addition, we provide a functional description and a brief description of the pathophysiology of some clinical considerations that may underlie ARSA, such as variants of the recurrent laryngeal nerve.^[71]^ In relation to the above, we have found 4 reviews that took a similar approach. It should be noted that based on our search, there is only 1 study that performed an ARSA meta-analysis,^[72]^ which is 8 years out of date with ours. Scala conducted a meta-analysis looking at the presence of ARSA in fetuses with Down syndrome, which showed that ARSA appears to be a clinically useful prenatal ultrasound marker of Down syndrome. Additional testing when ARSA is diagnosed should include evaluation of all risk factors by applying a mathematical model; Scala also proposed that there is insufficient evidence to recommend fetal karyotyping in cases with isolated ARSA, which is related to the relationship that we show in a limited way associated with ARSA and ARSA Down syndrome. In addition, it clarifies that our meta-analysis focuses on the morphological characteristics of ARSA and its clinical and surgical evolution. Another of the studies that met the criteria of this review is the one by Popieluszko et al,^[73]^ where he carried out a systematic review and meta-analysis of all the variants of the aortic arch. As a result, he showed that patients with an aortic arch variant are often asymptomatic, constitute a significant part of the patient population, and are at increased risk of bleeding and ischemia during chest surgery. Although this study shows the prevalence of ARSA, its evaluation is global of all the variants of the aortic arch, while our study focuses on a detailed analysis of the ARSA and its anatomoclinical considerations. Finally, in our search, we found the study by Natsis et al^[24]^ that analyzed the ARSA variations that are usually asymptomatic and can cause dyspnea, dysphagia, intermittent claudication, misinterpretation of radiological examinations, and complications during neck and thorax surgery. In addition, these variations may be accompanied by other congenital anomalies such as variants of other structures including the recurrent laryngeal nerve. In relation to the above, our study carried out a detailed and exhaustive analysis of the ARSA, including classifications according to the type of ARSA, risk of bias of the included studies, prevalence, and clinical consideration of the included studies.

The included studies were of various methodological types, but those used to analyze the prevalence and clinical characteristics of ARSA were case reports and retrospective or prospective observational studies. Regarding the characteristics of the sample, it was quite homogeneous between men and women; therefore, we cannot underestimate that the presence of ARSA is associated with the sex of the individual. Regarding the geographical distribution, the studies were performed in a homogeneous way between Asia, Europe, and North America, none of which showed that the presence of ARSA can be related to any specific type of ethnicity or race.

Among the articles that met our inclusion criteria, the average prevalence of ARSA was 2%, which is associated with an adequate theoretical prevalence for an anatomical variant and not anatomical modification. In relation to the above, ARSA can follow several routes, the most classic being the retroesophageal route; it could also have a route between the esophagus and the trachea, and, finally, it could run anterior to the trachea, the retroesophageal being the one described above, which is more frequently associated with clinical manifestations. In 5% of cases where retroesophageal aberrant subclavian artery was present, ARSA presents symptomatically and can be demonstrated as LD, supporting the theory proposed by this study that ARSA produces LD in low cases. But it is worth mentioning what was reported by Hegazy,^[5]^ which mentions that there may be a congenital ring of the aortic arch because the right dorsal aorta does not disappear distally, so that an arch can form surrounding the trachea and esophagus with possible dyspnea and dysphagia. In this case, the right subclavian artery arises from the involuntary vascular ring.^[5,6]^ From the above, the LD shows the following symptoms: dyspnea in 25%, chest pain in 16%, cough in 8%, and claudication of the corresponding upper limb in 5% of cases,^[8]^ which manifests in 10% of LD with ARSA associate. The symptoms of LD in most cases occur in people who are prone to developing atherosclerotic diseases and begin around 40 years of age. In 60% of cases, ARSA may be associated with a KD, an aneurysm that originates from the descending thoracic aorta. This KD may be relevant in surgical approaches to treat LD.^[8]^ Among the most serious complications associated with ASDA, aortic dissection, recurrent pneumonia, and obstructive emphysema are found.^[8,9]^ Knowledge of this anatomical variant is of great clinical and surgical interest, both in diagnostic and therapeutic procedures and as an antecedent for the investigation of some genopathies, aneupleudia, and cardiac or extracardiac congenital malformations. Regarding all these clinical considerations, it is important to understand that many times ARSA will be asymptomatic throughout life, but one should note that it is highly important to know its presentations since in the presence of symptoms associated with ARSA, it is crucial to make a good diagnosis and be able to have a good therapeutic or surgical approach. In the presence of these symptoms and when other possible more prevalent pathologies have been ruled out, it is important to perform imaging studies that allow the observation of ARSA. It is also suggested to study the patient when this anomaly is suspected due to previous ultrasound findings. Several studies recommend surgical management as the initial treatment to prevent complications derived from ARSA. Finally, the studies included in this meta-analysis presented a moderate and high risk of bias, which limits the data reported by this review to be taken with caution.

## 5. Limitations

The limitations of this review are the publication bias of the included studies since studies with different results that were in non-indexed literature in the selected databases may have been left out; the probability of the researchers not having carried out the most sensitive and specific search regarding the topic to be studied; and, finally, personal preference of the authors for the selection of articles.

## 6. Conclusion

The prevalence of ARSA in this study was quite low, beyond the 5% proposed by studies from a theoretical point of view. We believe that knowing the different types of origin and paths of ARSA is important in the development of medical professionals or other professionals who I know may encounter this variant; it is also important to understand that in the presence of ARSA, the classic pathology will not always be lustful dysphagia, but rather there are varied symptoms, for which we believe that a good knowledge for professionals who treat the cervical and thoracic region becomes crucial for a good diagnosis and treatment in the presence of ARSA.

## Author contributions

Conceptualization: Juan José Valenzuela-Fuenzalida, Mathias Orellana-Donoso, Daniela Perez-Jiménez, Emilio Farfán-Cabello, Marjorie Gold-Semmler, Alvaro Becerra-Farfan, Pablo Nova-Beza.

Data curation: Juan José Valenzuela-Fuenzalida, Mathias Orellana-Donoso, Daniela Perez-Jiménez, Marjorie Gold-Semmler.

Formal analysis: Juan José Valenzuela-Fuenzalida, Daniela Perez-Jiménez, Emilio Farfán-Cabello, Pablo Nova-Beza.

Funding acquisition: Juan José Valenzuela-Fuenzalida, Alvaro Becerra-Farfan.

Investigation: Juan José Valenzuela-Fuenzalida, Alvaro Becerra-Farfan, Camila Román, Pablo Nova-Beza.

Methodology: Juan José Valenzuela-Fuenzalida, Camila Román.

Validation: Juan José Valenzuela-Fuenzalida, Mathias Orellana-Donoso, Daniela Perez-Jiménez, Emilio Farfán-Cabello, Marjorie Gold-Semmler.

Visualization: Juan José Valenzuela-Fuenzalida, Mathias Orellana-Donoso, Daniela Perez-Jiménez, Emilio Farfán-Cabello, Alvaro Becerra-Farfan.

Writing—original draft: Juan José Valenzuela-Fuenzalida, Mathias Orellana-Donoso, Daniela Perez-Jiménez, Emilio Farfán-Cabello, Alvaro Becerra-Farfan.

Writing—review & editing: Juan José Valenzuela-Fuenzalida, Daniela Perez-Jiménez, Emilio Farfán-Cabello.

Resources: Daniela Perez-Jiménez, Camila Román, Pablo Nova-Beza.

Supervision: Daniela Perez-Jiménez, Marjorie Gold-Semmler, Camila Román.

Software: Marjorie Gold-Semmler, Camila Román, Pablo Nova-Beza.

Project administration: Camila Román, Pablo Nova-Beza.

## Supplementary Material




