# Supplementary material for: Systematic review and meta-analysis of right subclavian artery variants and their correlation with cervical-thoracic clinical conditions
Source: Medicine (Baltimore). 2024 Feb 23;103(8):e36856. doi: 10.1097/MD.0000000000036856 (PMC11309723; doi:10.1097/MD.0000000000036856)
Supplement: Supplementary file 1 [file medi-103-e36856-s001.docx]

**Supplemental Digital Content. Table S1.** Searches strategies

| Database | Search strategy | Results | |
| --- | --- | --- | --- |
|  |  | 07-06-23 |  |
| Medline | ((((((aberrant subclavian artery) OR (aberrant right subclavian artery)) OR (lusory artery)) AND (anatomical variations)) AND (clinical anatomy)) OR (dysphagia lusory)) OR (kommerell diverticulum)) NOT (animals) | 531 |  |
| SCOPUS | Aberrant subclavian artery) OR (aberrant right subclavian artery)) OR (lusory artery)) AND (anatomical variations)) AND (clinical anatomy)) OR (dysphagia lusory)) OR (kommerell diverticulum)) NOT (animals) | 123 |  |
| Google scholar | Aberrant subclavian artery) OR (aberrant right subclavian artery)) OR (lusory artery)) AND (anatomical variations)) AND (clinical anatomy)) OR (dysphagia lusory)) OR (kommerell diverticulum)) | 231 |  |
| CINHAL | Aberrant subclavian artery) OR (aberrant right subclavian artery)) OR (lusory artery)) AND (anatomical variations)) AND (clinical anatomy)) OR (dysphagia lusory)) OR (kommerell diverticulum)) | 42 |  |
| WOS | Aberrant subclavian artery) OR (aberrant right subclavian artery)) OR (lusory artery)) AND (anatomical variations)) AND (clinical anatomy)) OR (dysphagia lusory)) OR (kommerell diverticulum)) | 345 |  |
|  | Total | 1272 |  |

* All searches were carried out on May 27, 2023.
