# Supplementary material for: Systematic review and meta-analysis of right subclavian artery variants and their correlation with cervical-thoracic clinical conditions
Source: Medicine (Baltimore). 2024 Feb 23;103(8):e36856. doi: 10.1097/MD.0000000000036856 (PMC11309723; doi:10.1097/MD.0000000000036856)
Supplement: Supplementary file 2 [file medi-103-e36856-s002.docx]

**Supplementary Table 2: excluded studies and reason.**

| Study | Type of study | Reason for exclusion |
| --- | --- | --- |
| Zainali-Gill & Buren, 2015 | Case report | It was excluded because the case report did not provide relevant or novel information for the investigation. |
| Hart & Kamath, 2012 | Case report | It was excluded because the case report did not provide relevant or novel information for the investigation. |
| Lam et al., 2019 | Case report | It was excluded because the case report did not provide relevant or novel information for the investigation. |
| Chudasama & Alcorn, 2016 | Case report | It was excluded because the case report did not provide relevant or novel information for the investigation. |
| Hudzik & Gąsior, 2016 | Case report | It was excluded because the case report did not provide relevant or novel information for the investigation. |
| Mandavdhare, 2018 | Case report | It was excluded because the case report did not provide relevant or novel information for the investigation. |
| Koop & Francis, 2019 | Case report | It was excluded because the case report did not provide relevant or novel information for the investigation. |
| Soo Hoo et al., 2018 | Case report | It was excluded because the case report did not provide relevant or novel information for the investigation. |
| Alourfi & Mosli, 2020 | Case report | It was excluded because the case report did not provide relevant or novel information for the investigation. |
| de Araújo et al., 2015 | Case report | It was excluded because the case report did not provide relevant or novel information for the investigation. |
| Jalal et al., 2013 | Case series | It was excluded because the case report did not provide relevant or novel information for the investigation. |
| Thompson & Bukhart, 2016 | Case report | It was excluded because the case report did not provide relevant or novel information for the investigation. |
| Gnanapandithan et al., 2014 | Case report | It was excluded because the case report did not provide relevant or novel information for the investigation. |
| Sigdel et al., 2020 | Case report | It was excluded because the case report did not provide relevant or novel information for the investigation. |
| Rathnakar et al., 2014 | Case report | It does not explain in detail the variant of the ARSA |
| Fernández Álvarez et al., 2020 | Case report | It does not explain in detail the variant of the ARSA |
| De Caluwe et al., 2012 | Case report | It does not explain in detail the variant of the ARSA |
| Lesko et al., 2014 | Case report | It does not explain in detail the variant of the ARSA |
| Irakleidis et al., 2020 | Case report | It does not explain in detail the variant of the ARSA |
| Fukuhara et al., 2012 | Case report | It does not explain in detail the variant of the ARSA |
| Venugopal et al., 2012 | Case report | It does not explain in detail the variant of the ARSA |
| Leite et al., 2017 | Case report | It does not explain in detail the variant of the ARSA |
| Debonnaire et al., 2012 | Case report | It does not explain in detail the variant of the ARSA |
| Tuleja et al., 2019 | Case report | It does not explain in detail the variant of the ARSA |
| Okumus et al., 2014 | Case report | It does not explain in detail the variant of the ARSA |
| Mittal et al., 2012 | Case report | It does not explain in detail the variant of the ARSA |
| La Regina et al., 2020 | Case report | It does not explain in detail the variant of the ARSA |
| González-Sánchez et al., 2013 | Case report | It does not explain in detail the variant of the ARSA |
| Derbel et al., 2012 | Case report | It does not explain in detail the variant of the ARSA |
| Rogers et al., 2011 | Case report | It does not explain in detail the variant of the ARSA |
| Cobos et al., 2016 | Case report | It does not explain in detail the variant of the ARSA |
| Breaux et al., 2014 | Case report | It does not explain in detail the variant of the ARSA |
| Barone et al., 2016 | Case report | It does not explain in detail the variant of the ARSA |
| Reynolds et al., 2015 | Case report | It does not explain in detail the variant of the ARSA |
